# Fulminant Giant Cell Myocarditis and Cardiogenic Shock: An Unusual Presentation of Malignant Thymoma

**DOI:** 10.4061/2010/185896

**Published:** 2010-05-04

**Authors:** Deepak Koul, Manreet Kanwar, Dane Jefic, Anuradha Kolluru, Tejwant Singh, Sunil Dhar, Preetham Kumar, Gerald Cohen

**Affiliations:** ^1^Division of Cardiology, Cardiac Cath Laboratory, St. John Hospital and Medical Center, 22101 Moross Road, 2nd Floor VEP, Detroit, MI 48236, USA; ^2^St. John Hospital and Medical Center, 22101 Moross Road, Detroit, MI 48236, USA; ^3^Abington Memorial Hospital, Drexel University College of Medicine, 1200 Old York Raod, Abington, PA 19001, USA

## Abstract

Malignant thymoma is rarely associated with giant cell myocarditis. We present a case study that illustrates this association and cardiogenic shock with underlying tamponade. The dramatic presentation of this scenario has not been previously described.

## 1. Introduction

Giant cell myocarditis (GCM) is a rare and frequently fatal type of myocarditis. Patients usually die of heart failure and ventricular arrhythmia unless cardiac transplantation is performed [1]. In addition to the idiopathic or primary form, GCM has been associated with other conditions including autoimmune diseases, granulomatosis, and neoplastic conditions [2]. However, the etiology and pathogenesis of GCM is not well known. 

 Very few cases of malignant thymoma with GCM have been reported. We describe a case of GCM with dramatic clinical presentation of cardiac tamponade and cardiogenic shock.

## 2. Case Report

**Figure 1:**
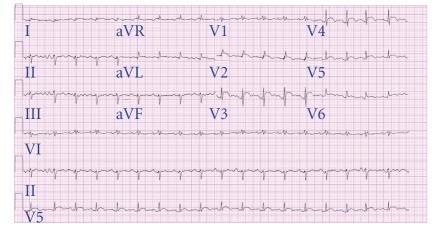
Electrocardiogram showing ST segment elevation in leads I, aVL, and V2–V6.

A previously healthy 55-year-old woman presented with one-week history of worsening muscle weakness, diplopia, and exertional dyspnea. Physical examination revealed a pale thin female with tachycardia (107 beats/minute), tachypnea (22 breaths/minute), hypotension (blood pressure 86/50 mmHg), jugular venous distension with rapid “×” descent, and distant heart sounds. While the patient was being evaluated in the emergency room, she suddenly had a cardiopulmonary arrest. After resuscitation, an echocardiogram revealed severe left ventricular (LV) dysfunction with cardiac tamponade. Emergent pericardiocentesis was done and 50 mL of pericardial fluid was drained. An extra-pericardial mass compressing the left atrium was also suspected, but images were limited. Electrocardiogram showed 1 mm ST segment elevation in anterior chest leads (Figure 1). Initial labs revealed elevated troponin I (8.38 ng/mL) and creatine kinase-MB (21 ng/mL). Since this suggested possible acute myocardial infarction, she was emergently taken for cardiac catheterization, but no coronary artery disease was detected. She was hemodynamically sustained with inotropes and an intraaortic balloon pump (IABP). 

**Figure 2:**
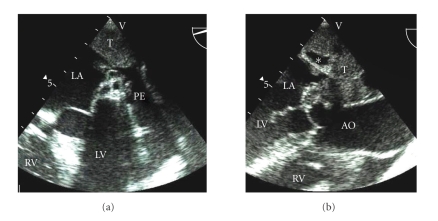
(a) Transesophageal echocardiogram of 4-chamber view showing tumor mass (T) compressing the left atrium (LA). A pericardial effusion (PE) is adjacent to the left ventricular (LV) free wall. RV = right ventricle. (b) Transesophageal echocardiogram of left ventricular (LV) outflow tract in long axis showing homogenous tumor (T) mass with lucent spaces (∗) compressing the left atrium (LA). AO = Ascending aorta. RV = right ventricle.

**Figure 3:**
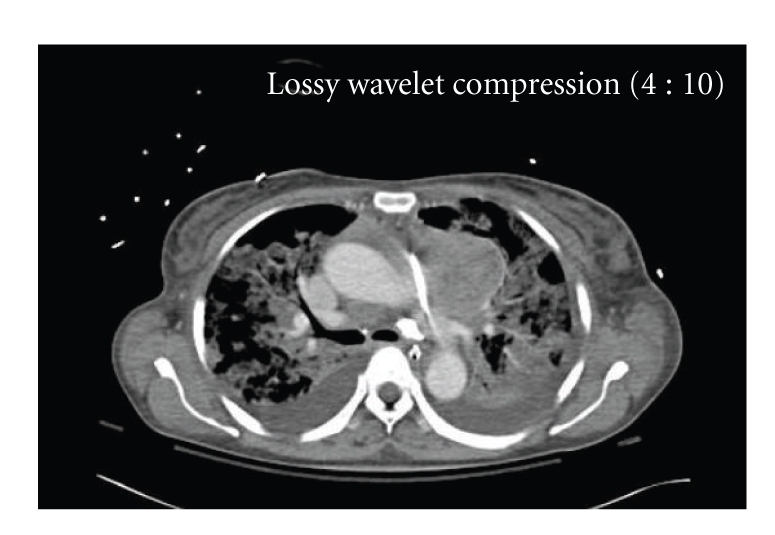
Computed axial tomogram showing a mass in left anterior mediastinum at the root of pulmonary artery with right anterior pericardial involvement and effusion.

 Transesophageal echocardiogram showed global LV severe hypokinesis with ejection fraction (EF) of 10 percent and a large mass indenting the left atrium and pulmonary veins (Figures 2(a) and 2(b)). On CT scan, a large superior mediastinal mass extended to the aortopulmonary window and abutted the aortic arch and pulmonary artery (Figure 3). CT guided biopsy revealed thymoma with undifferentiated cells. 

**Figure 4:**
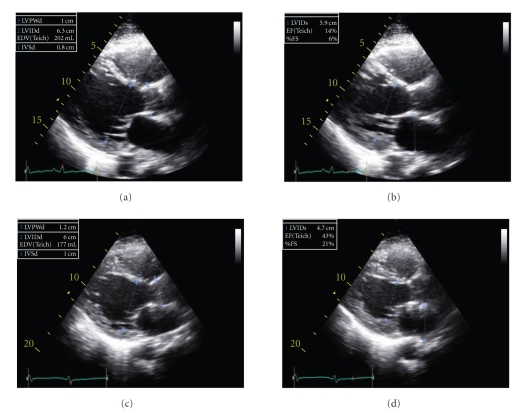
Parasternal long-axis views showing severe baseline LV dysfunction (Figures 4(a) and 4(b)) and improved ejection fraction on the followup study (Figures 4(c) and 4(d)).

 Further laboratory evaluation showed elevated acetylcholine receptor antibody, antisarcolemma, and antimyosin antibody titers. Antibody titers of viral etiologies for myocarditis were negative. Steroids and plasmapheresis for thymoma-associated myasthenia gravis resulted in neurological improvement. Patient was taken off of inotropes and IABP. A repeat echocardiogram several days later showed improved LV function with an EF of 45% (Figure 4). Chemotherapy with carboplatin and paclitaxel resulted in good initial response with CT scan showing reduction in thymoma tumor burden. However, hospital course was complicated by febrile neutropenia and sepsis. She died 35 days after hospital admission. 

**Figure 5:**
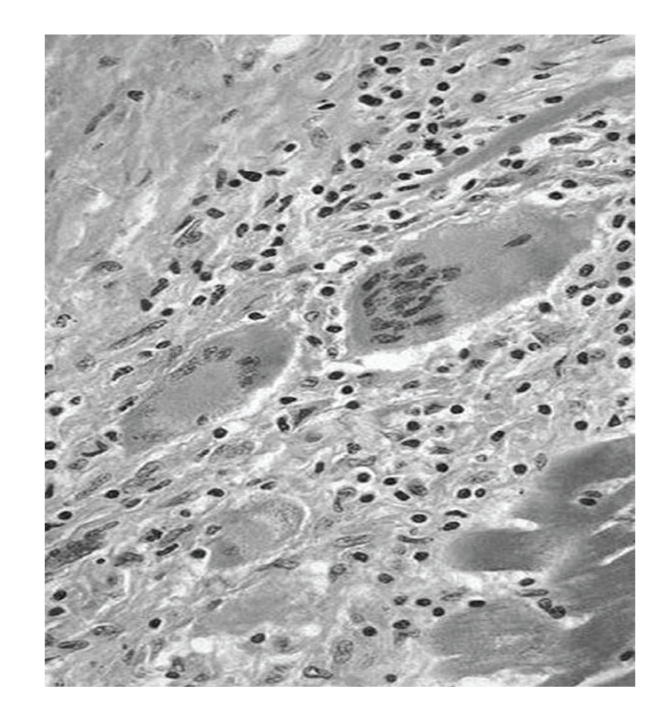
Histopathology demonstrating multinucleated giant cells and numerous lymphocytes infiltrating the cardiomyocytes.

On autopsy, the heart weighed 439 grams and was uniformly soft and pale without coronary obstruction or valvular abnormality. Histological examination of the myocardium showed large multinucleated epitheliod cells, plasma cells, and lymphocytes characteristic of GCM (Figure 5).

## 3. Discussion

GCM has a rare association with malignant thymoma with or without clinical manifestations of myasthenia gravis [2–4]. Pathogenesis is unknown and prognosis is very poor. In an animal model, a disorder similar to GCM was induced by immunization with cardiac myosin [5]. Myasthenia gravis is an autoimmune disorder and is found to be associated with thymoma in about 15% of the cases. Association of giant cell myocarditis with thymoma, myasthenia gravis, and other autoimmune disorders suggests that it may be another manifestation of “immune dysregulation”. In this case report, myasthenia gravis was confirmed by finding high titers of acetylcholine receptor antibodies. Other nonspecific antibodies like antisarcolemma and antimyosin antibodies provided suggestive evidence that GCM is an autoimmune disease of the myocardium. 

 Presentation and clinical course of GCM is usually characterized by rapid deterioration in LV function, frequent ventricular arrhythmias, and heart block [1]. In our patient, cardiac tamponade and cardiogenic shock was secondary to underlying GCM. Our patient had fulminant presentation; however, no significant ventricular arrhythmia was seen. 

 Unlike other types of myocarditis, GCM has been shown to respond to immunosuppressive therapy with significant improvement in survival [1, 6]. Improvement in LV function in our patient may have been due to immunosuppressive drug treatment, though she died because of sepsis. Autopsy confirmed GCM and its rare association with malignant thymoma.

## 4. Conclusion

This case illustrates the unusual association of thymoma and myasthenia gravis with fulminant myocarditis. To our knowledge, thymoma-associated GCM presenting as cardiac tamponade and cardiogenic shock has not been reported. Myocarditis was likely due to autoantibodies. Immunosuppressive therapy was associated with improved LV function, but ultimately fatal sepsis. This case also highlights the systemic nature of malignant thymoma.
